# Simulating Partial Conduction Delays in VEPs Via Asynchronous and Contrast-Modulated Stimulation

**DOI:** 10.1167/tvst.15.9.5

**Published:** 2026-09-08

**Authors:** Leon A. Pfeiffer, Julia Haldina, Sven P. Heinrich

**Affiliations:** 1 Eye Center, Medical Center, University of Freiburg, Freiburg, Germany; 2 Faculty of Medicine, University of Freiburg, Freiburg, Germany; 3 Faculty of Biology, University of Freiburg, Freiburg, Germany

**Keywords:** visual evoked potentials, conduction delay, double peaks, modelling, optic neuritis

## Abstract

**Purpose:**

To investigate the origin of P100 double peaks occasionally observed in patients with optic neuritis by experimentally inducing timing asynchronies between visual field locations and modulating stimulus strength via contrast reduction in transient and steady-state VEPs (trVEP and ssVEP).

**Methods:**

The trVEPs and ssVEPs were recorded using a checkerboard stimulus vertically split into nasal and temporal half-fields. Timing asynchrony was induced by delaying the temporal half-field by 24, 35, or 47 ms; stimulus strength was modulated by reducing the temporal half-field contrast to 20% or 8%. Full-field and half-field responses were quantitatively analyzed and qualitatively compared to characterize the superposition effects induced by these stimulus changes.

**Results:**

In trVEPs, contrast reduction produced smaller, broader P100 responses with modest peak-time delays and only sporadic double-peak formation. Conversely, induced temporal half-field delays robustly generated P100 double peaks (incidence: 50% at 24 ms, 85% at 35 ms, and 100% at 47 ms). Quantitative and qualitative analyses demonstrated that the P100 peaks of the half-field responses aligned temporally with the two peaks of the full-field double peak. In ssVEPs, increasing delays reduced the absolute harmonic magnitude and altered the second-to-first harmonic ratio (decreased at 24 and 35 ms; increased at 47 ms).

**Conclusions:**

Spatially localized timing asynchronies reliably reproduce double-peaked P100 morphologies, supporting differential conduction speeds within the optic nerve as a plausible mechanism in patients with optic neuritis.

**Translational Relevance:**

Linking experimentally induced conduction-delay effects to VEP morphology enhances the clinical interpretation of atypical VEP waveforms in suspected optic neuritis.

## Introduction

The visual evoked potential (VEP) evaluates the functional integrity of the visual pathways up to the primary visual cortex (V1) in the occipital lobe.^1^ In routine clinical applications, it is obtained using a checkerboard stimulus (pattern-reversal or onset/offset).^2^ Two variants are frequently employed to aid the clinical evaluation of patients. The transient VEP (trVEP) is used in the diagnosis of potential compressive or demyelinating pathologies affecting the optic nerve by measuring amplitude and peak time of the P100 deflection. This includes suspected optic neuritis (ON), with peak time delay as a key indicator.^3^ The steady-state VEP (ssVEP) is frequently used in the objective estimation of visual acuity. As such, it has gained increasing interest, for instance, in cases of unexplained visual loss and malingering.^4^

**Figure 1.**
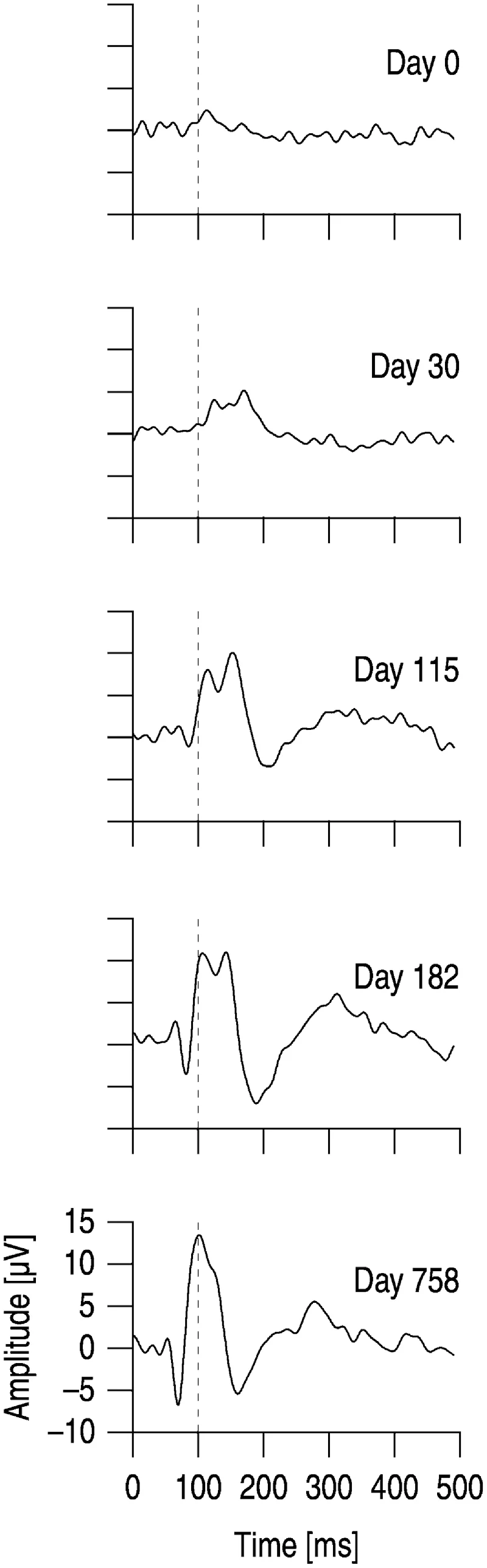
VEP recordings from the affected eye of a patient with optic neuritis followed up for two years. Initially, almost no response was present. On Day 30, a relatively broad deflection of small amplitude had manifested, which was crowned by several peaklets. By Day 115, amplitude was in the normal range, and two distinct subpeaks had formed. Subsequently, the peak time of the earlier subpeak gradually normalized while the peak time of the second subpeak shortened even more, causing it to merge towards the first subpeak. By Day 758, the second subpeak merely appears as a shoulder on the trailing flank of the first subpeak. (Data from the TONE clinical trial.^7^)

A prerequisite for obtaining reliable amplitude and peak time readings from the trVEP is a sufficiently well-defined P100 peak. Occasionally, a double-peaked P100 is encountered in clinical routine testing^5^^,^^6^ (Fig. 1), presenting a conundrum for the analysis and raising questions regarding the origin of this waveform.

Various physiological and morphological factors could, in principle, give rise to double peaks, as detailed in the discussion. Regarding optic neuritis, building on the principle of superposition and observations of temporally variable double-peaked P100 waveforms in the optic neuritis cohort (30 days to two years post-onset), asynchronous myelination patterns have been proposed as an underlying mechanism. In this model, a subset of optic nerve fibers exhibits a slower conduction velocity than others.^8^ An initial experimental study simulated partial conduction delays by reducing the luminance of half the stimulation screen and investigated the resulting effects on waveforms.^8^ This method was based on the mechanisms underlying the perceptual Pulfrich effect^9^ and assumes a slowed conduction velocity of the fibers responsible for the retinal area stimulated by reduced luminance. The study highlighted limitations of P100 amplitude and peak time measurements in detecting pathological states of the optic nerve, although no systematic double-peak formation or other definitive waveform changes were identified to support the clinical analysis of such cases.

In the present study, we explored the generation of double-peaked P100 responses by experimentally inducing timing differences in neuronal responses between visual field locations. In addition, we investigated the effects of amplitude variations by altering stimulus strength through the adjustment of contrast. These manipulations were applied to both trVEPs, given their role in the diagnosis of ON, and in ssVEPs. Although ssVEPs are not routinely used for the diagnosis of ON, they are applied in other clinical contexts, such as objective estimation of visual acuity, where responses are commonly analyzed in the frequency domain.^4^ We therefore included ssVEPs to assess whether partial conduction delays may alter spectral response composition, particularly the relative contribution of the first and second harmonics.

## Methods

### Participants

The trVEPs and ssVEPs were evaluated in separate experiments, each including 20 healthy participants (see Supplementary Table S1 for details on the study populations) who provided written informed consent. The study was part of a larger project approved by the institutional review board and followed the tenets of the Declaration of Helsinki. All participants reported no ophthalmologic, neurologic, or psychiatric conditions. Visual acuity (VA) and contrast sensitivity (CS) were measured using the semi-automated Freiburg Visual Acuity and Contrast Test^10^ to ensure that the inclusion criteria of VA ≤0.0 logMAR and CS ≥1.6 logCS were met. All subsequent data were acquired monocularly, using the participant's sighting-dominant eye whenever feasible and with pupils in natural miosis.

### Stimulation

Consistent with the guidelines of the International Society for Clinical Electrophysiology of Vision (ISCEV) concerning the trVEP^11^ and ssVEP,^12^ an EIZO FlexScan F57 CRT monitor with a refresh rate of 85 Hz was used at an eye-to-screen distance of 114 cm. Stimuli subtended a visual angle of 16° × 13° (height × width). Illuminance at the participant's eye level was 680 lux. Stimuli were created using PsychoPy 3,^13^ with timing accuracy verified via photosensor and luminance/contrast levels confirmed by a luminance meter.

Two check sizes of 1.00° (characteristic spatial frequency [SF] = 0.7 cpd) and 0.25° (SF = 2.8 cpd) with a mean screen luminance of 60 cd/m^2^, Michelson contrasts of 94%, 20%, and 8%, and a reversal rate of 2.02/s were used for the trVEP. The ssVEP was recorded using onset-offset stimulation at 7.73 Hz; patterns were presented for 47.1 ms, interleaved with a homogenous gray field of the same average luminance presented for 82.4 ms. In addition to the trVEP check sizes, a third size of 0.06° (SF = 11.8 cpd) was included. The luminance was the same as for the trVEP, except for the 0.06°-sized checks, where technical constraints imposed by the CRT monitor limited the mean luminance to 55 cd/m^2^.

**Figure 2.**
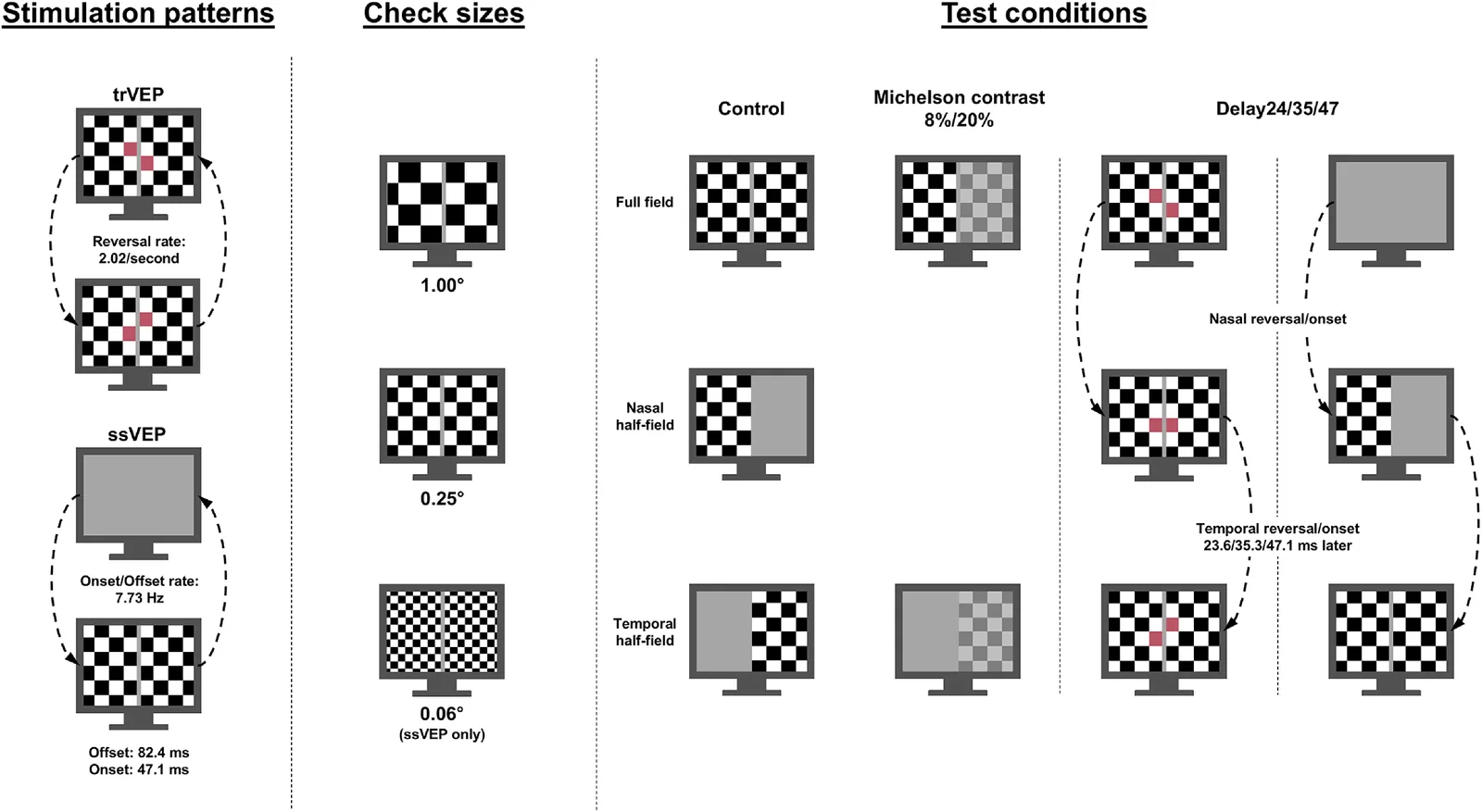
Experimental setup exemplified for monocular stimulation of the right eye. The trVEP was obtained with black-and-white checkerboard patterns reversing at a rate of 2.02/s; the ssVEP was recorded to black-and-white checkerboard patterns appearing at a rate of 7.73 Hz from a homogenous gray background. Stimulus duration was 47.1 ms, the interstimulus interval was 82.4 ms. Two check sizes of 1.00° and 0.25° were used for the trVEP, with an additional third one of 0.06° included for the ssVEP. Test conditions comprised full-field, temporal and nasal half-field stimulation with 94% contrast patterns (control conditions), full-field stimulation with the temporal half-field at 8% or 20% contrast, and temporal half-field stimulation at 8% or 20% contrast (contrast conditions), and full-field stimulation with the temporal half-field reversal/onset being delayed by 23.5, 35.3, or 47.1 ms (delay conditions). Red checkers in the reversal stimuli are colored for visualization of the pattern reversal only. From top to bottom, the respective panels show the sequence of reversals. Fixation mark not shown.

For the experiment, the checkerboard pattern was vertically split in half by a 0.25°-wide grey bar, and 10 conditions were created (Fig. 2):

I. Control conditions (high (94%) contrast)
  1. Full-field stimulation (no asynchrony between half-fields)
  2. Nasal half-field stimulation
  3. Temporal half-field stimulation
II. Low-contrast conditions (no asynchrony)
  4. Full-field stimulation with the temporal field at 8% contrast
  5. Temporal half-field stimulation at 8% contrast
  6. Full-field stimulation with the temporal half-field at 20% contrast
  7. Temporal half-field stimulation at 20% contrast
III. Delay conditions (high contrast)
  8. Delay24: temporal half-field reversal/onset 23.5 ms (2 frames) delayed
  9. Delay35: temporal half-field reversal/onset 35.3 ms (3 frames) delayed
  10. Delay47: temporal half-field reversal/onset 47.1 ms (4 frames) delayed

A central fixation target of 0.25° was displayed. To ensure appropriate fixation, accommodation, and attention, a random number between 1 and 9 was briefly presented overlaid on the central fixation target at random intervals for the participant to report out loud. The presentation order of the 10 conditions was individually randomized for each participant.

#### EEG Acquisition and Analysis

EEG recordings were performed with gold-cup electrodes. Impedances were maintained below 5 kΩ throughout the measurements. The trVEP was recorded with the active electrode on the Oz position as defined by the 10-10 system,^14^ with the reference electrode at Fpz and the ground electrode attached to an earlobe. The ssVEP was recorded additionally from the O1 and O2 positions to compute a virtual Laplacian channel as Oz − (O1+O2)/2.^12^ The EEG was acquired with a BrainAmp EEG system (Brain Products, Gilching Germany). Signals were band-pass filtered at 0.2–120 Hz, 50,000-fold amplified, and sampled at a rate of 500 Hz. An artifact rejection threshold of ±100 µV was applied.

As recommended by the ISCEV guidelines, each condition was recorded twice for the trVEP to confirm repeatability.^11^ 10 consecutive reversals were presented before switching to the next check size. This was continued until 100 artifact-free trials were recorded. For the ssVEP, data were recorded in a single block per condition. Eighty consecutive onset/offset stimulations were presented before switching to the next check size. This cycle was repeated until at least 80 artifact-free sweeps of 1.04 s duration (each encompassing eight stimulus onsets) had been collected.

All offline analyses were performed using custom software written for Igor Pro 8 (WaveMetrics Inc., Lake Oswego, OR, USA). A digital 40-Hz low-pass filter was applied to the EEG traces during the analysis. The first stimulation interval after each check size switch was discarded to preclude signal contamination arising from the transitions. One hundred artifacts-free trials were averaged for the trVEP and 80 sweeps were averaged for the ssVEP. To determine the noise-corrected magnitudes of the first and second harmonic of the ssVEP, Fourier analysis with noise correction, as outlined by Bach and Meigen,^15^ was applied to the averaged data.

**Figure 3.**
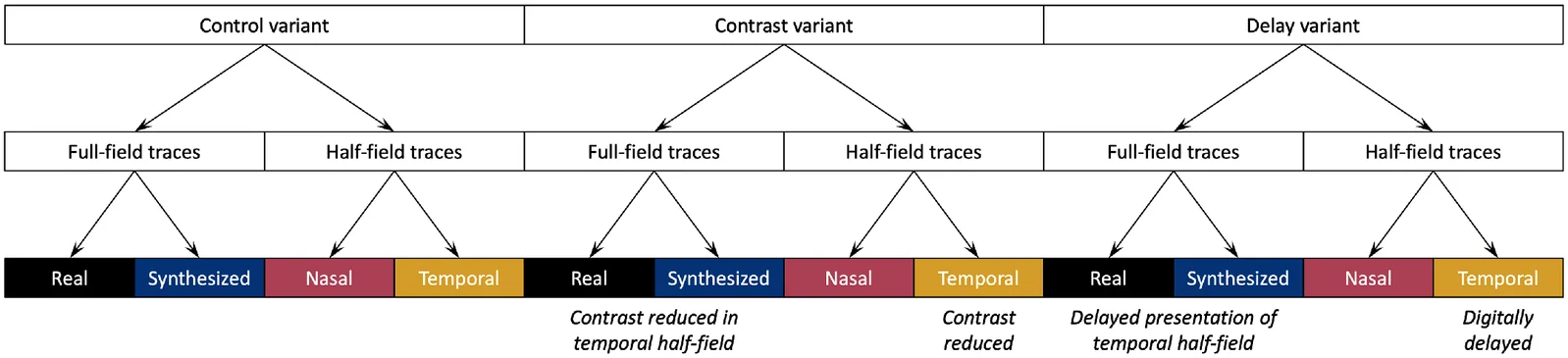
Overview of VEP traces included in the analysis. Synthesized traces were obtained by additive superposition of the half-field responses of the respective variant. The color coding introduced here is also used in subsequent figures showing full-field and half-field traces.

For all full-field conditions that were recorded, corresponding synthesized waveforms were computed from responses obtained in the half-field control conditions. Because of the sampling rate of 500 Hz, half-field delays were set to be 24, 36, and 48 ms. Figure 3 presents all traces included in the subsequent analysis.

#### Statistical Analysis

For the trVEP, peak times of N75, P100, and N135 were determined relative to stimulus reversal. P100 amplitude was defined as the peak-to-trough difference between the N75 and P100. A repeatability coefficient (RC) and a coefficient of variation (CoV)^16^ were calculated to evaluate repeated measurements. Additionally, a Bland-Altman analysis was performed to estimate the bias of the mean difference for P100 peak time and amplitude between real and synthesized traces.^17^ P100 peak time and amplitude for the contrast and delay conditions were compared to the control recordings by bootstrapping with 10,000 samples to determine 95% confidence intervals (CIs); statistical significance was assessed using a permutation test.^18^ Double-peak and shoulder incidence rates were determined and the inter-peak intervals of double peaks (P100 peak 2 − P100 peak 1) were evaluated. A double peak was defined as a P100 complex with two clearly distinguishable positive peaks (or peaklets), each forming a local positive maximum relative to the adjacent waveform segments. This operational definition was intentionally broader than the definition of a bifid waveform used in some previous publications, as it also includes cases of separated positive peaks that do not necessarily meet more restrictive criteria for “traditional” bifid morphology.^6^ A shoulder was defined as a secondary, non-peaking inflection point on the flank of the P100, characterized by a temporary relative flattening of the waveform's amplitude change. (cf., Fig. 1, Day 758). Qualitative morphological changes and characteristics of the VEPs in the contrast and delay conditions were assessed by comparing real and synthesized full-field traces to the respective full-field control traces. To elucidate the origins of these changes, the half-field traces were superimposed on their respective full-field traces in the figures to facilitate a comparison of the timing of the individual spatial components.

For the ssVEP, the magnitudes of the first and second harmonics of the Oz and the virtual Laplacian channels were determined. A Bland-Altman analysis was then applied to the first harmonic. The magnitude of the first harmonic, as well as the second-to-first harmonic, ratio of the contrast and delay conditions were quantitatively compared to the control conditions using the same statistical approach described for the trVEP experiment. Qualitative analysis entailed the same steps as described for the trVEP.

## Results

### Transient VEP

**Figure 4.**
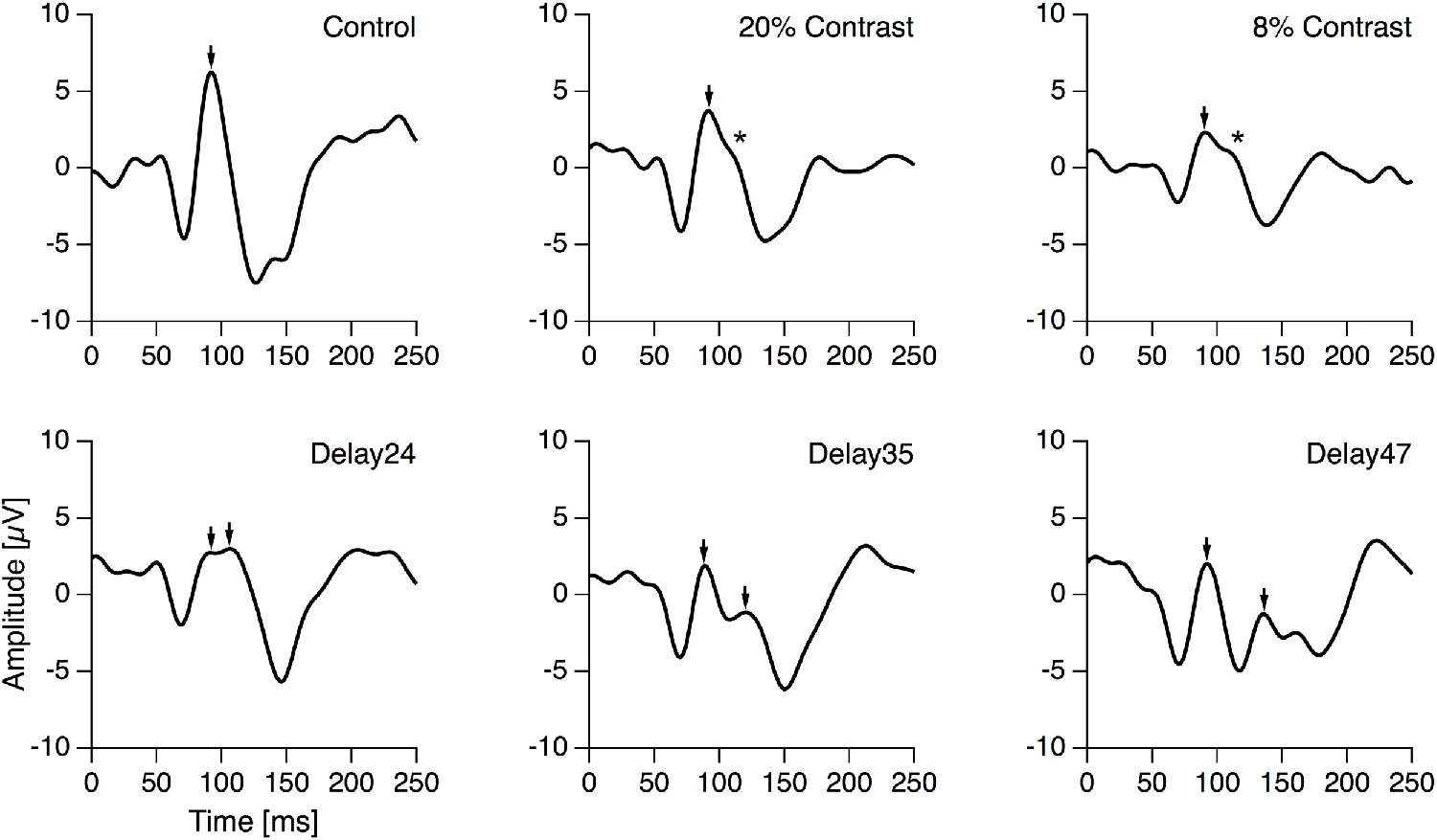
Example traces of one participant for the synchronous full-contrast full-field control condition and the conditions with either reduced contrast or asynchronous stimulation. In the low-contrast conditions, a shoulder (*asterisks*) developed on the descending flank of the P100. The 24-ms asynchrony resulted in a plateau with two small but distinct peaklets. With the 35 ms delay, a clear double peak emerged, which evolved into a distorted M-shaped configuration with the 47 ms delay. P100 peaks, including the individual subpeaks of the double peaks, are marked with arrows.

Figure 4 displays a representative set of VEP traces for the 0.25° full-field conditions from a single participant. Progressive contrast reduction manifested as a reduction of the P100 amplitude, a broadening of the peak morphology, and the development of a shoulder on the descending flank of the peak. The bottom graphs depict the delay conditions, in which a progressively more pronounced double-peak emerged with increasing inter-peak intervals. Unless stated otherwise, only the results of the 0.25° traces will be shown in the following paragraphs, as differences from the 1.00° traces were negligible.

#### Control Conditions

Mean P100 peak time for the real and synthesized full-field control conditions were 93.4 ms (95% CI, 92.0–94.8) and 92.7 ms (95% CI, 91.4–94.0), respectively. Mean P100 amplitudes were 12.8 µV (95% CI, 11.7–14.5) and 13.0 µV (95% CI, 11.1–15.0), respectively. Mean P100 peak time and amplitude of the nasal half-field were 92.6 ms (95% CI, 91.2–94.2) and 6.5 µV (95% CI, 5.5–7.7), respectively. Nasal and temporal half-field traces of the control conditions were similar in waveform and demonstrated negligible differences in P100 peak time (Δ = +0.5 ms; 95% CI, −1.3 to 2.1; *P* = 0.65) and amplitude (Δ = +0.4 µV, 95% CI, −1.3 to 0.5; *P* = 0.37).

Repeated full-field measurements demonstrated low variability in peak time (RC = 3.7 ms) and moderate variability in amplitude (CoV = 15%) for the full-field control conditions. Likewise, Bland Altman analysis revealed good agreement between real and synthesized data for both peak time (Δ = 0.7 ms; 95% CI, −0.3 to 1.6) and amplitude (Δ = −0.2 µV; 95% CI, −1.1 to 0.8; Supplementary Fig. S1).

#### Contrast Conditions

**Figure 5.**
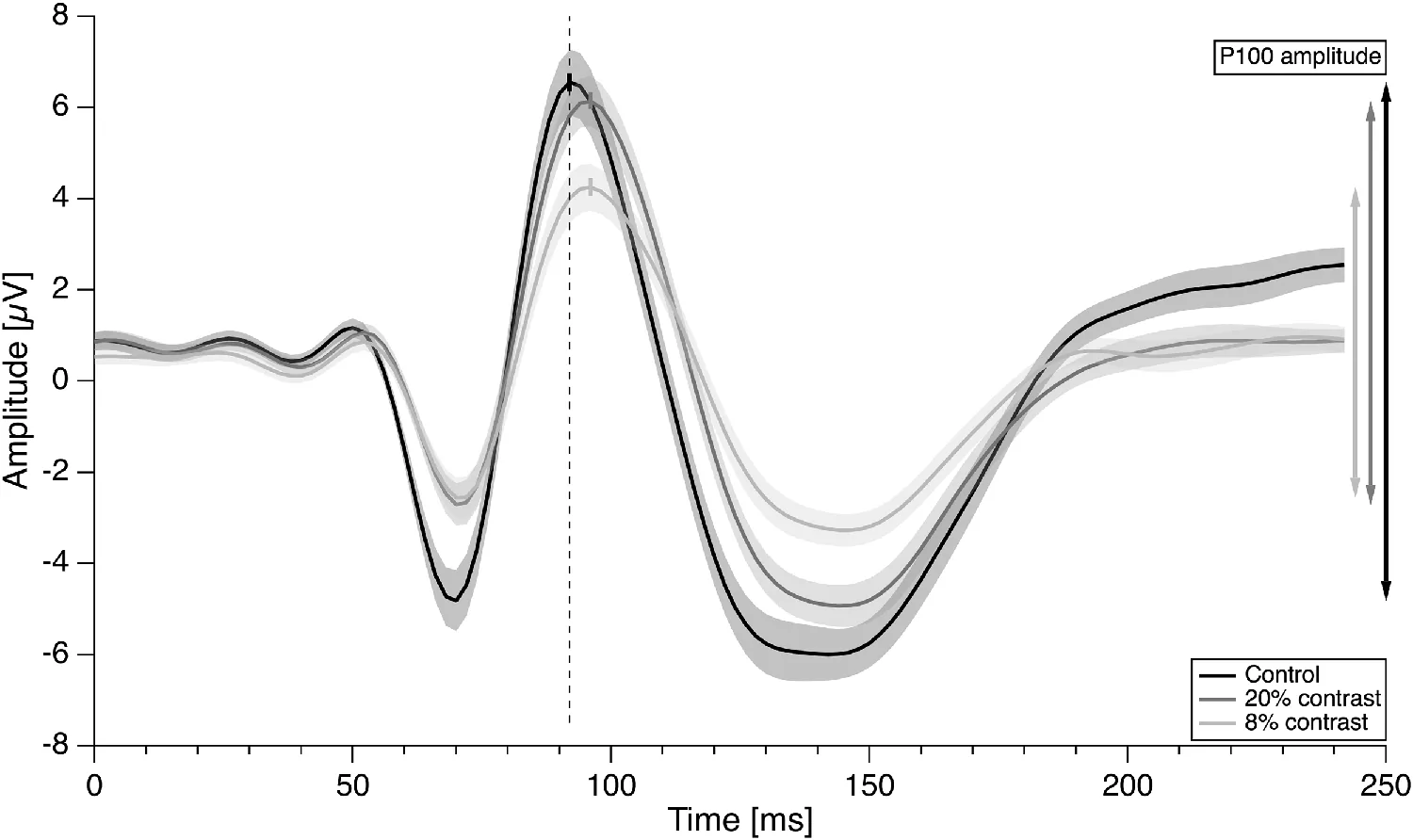
Grand mean (*n* = 20) full-field VEP traces of the 8% (*light* *gray* *line*) and 20% (*mid-gray line*) low contrast conditions, and the full contrast control condition (*black line*) of the 0.25° checks. Shaded areas represent the standard error of the mean (SEM). The *dashed vertical line* marks P100 peak time of the full contrast condition. P100 amplitudes (N75–P100 differences) at each contrast are displayed as corresponding arrows on the right side of the figure. With decreasing contrast, an increase of P100 peak time, a progressive decrease of P100 amplitude, and a broadening of the VEP complex occur.

To ensure accurate analysis of P100 peak time and amplitude, VEP traces with double peaks (see below) were excluded from the quantitative full-field analysis. Mean P100 peak time delays for the full-field 8% and 20% contrast conditions relative to the control were 5.1 ms (95% CI, 3.0–7.4; *P* < 0.001) and 3.0 ms (95% CI, 1.8–4.1; *pP* < 0.001), respectively. Relative to the control, mean P100 amplitudes reduced to 62% (−4.9 µV; 95% CI, −5.7 to −4.0; *P* < 0.001) and 75% (−3.1 µV; 95% CI, −3.9 to −2.5; *P* < 0.001), respectively. Qualitative analysis of both individual and grand mean traces revealed a progressive broadening of the P100 deflection, in addition to the time delay and amplitude reduction (Fig. 5).

**Figure 6.**
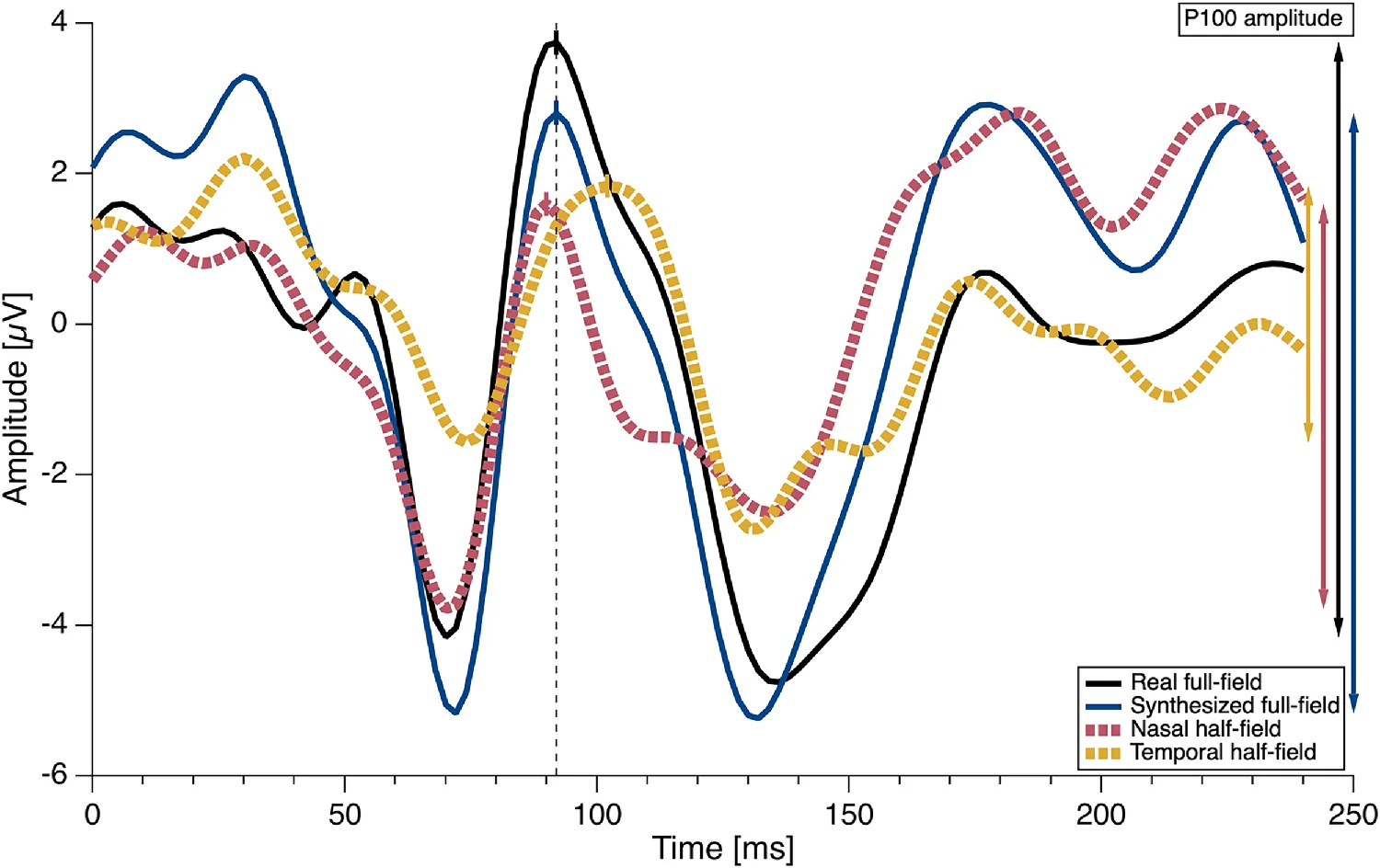
Example trace from one participant showing the 20%-contrast condition: full-fields (20%/100% half-fields), nasal half-field (100%), and temporal half-field (20%) responses. The real (*solid black line*) and synthesized (*solid blue line*) full-field traces exhibit a broadening waveform with characteristic shoulder development. The temporal half-field trace (*dashed yellow line*) displays a reduced P100 amplitude, a delayed peak time, and a broader deflection with a flattened peak compared to the nasal half-field trace (*dashed red line*).

Half-field responses from the contrast-reduced temporal field exhibited mean P100 peak time delays of 17.1 ms (95% CI, 14.9–19.3; *P* < 0.001) and 8.1 ms (95% CI, 5.9–10.0; *P* < 0.001) for the 8% and 20% contrast conditions, respectively. Figure 6 illustrates how these half-field responses with differential contrast summate to yield the resulting full-field response.

Double peaks appeared only sporadically in the conditions with differential contrast (1 out of 20 participants with 8% contrast, and none at 20% contrast), whereas shoulders, such as those seen in Figure 5, were more frequent with 8% contrast (four out of 20) than with 20% contrast stimulation (two out of 20). No check-size dependence was observed. Overall, qualitative analysis of the traces from the contrast condition revealed rather a pattern of gradual amplitude reduction and broadening of the waveform, accompanied by a modest peak time delay.

#### Delay Conditions

**Figure 7.**
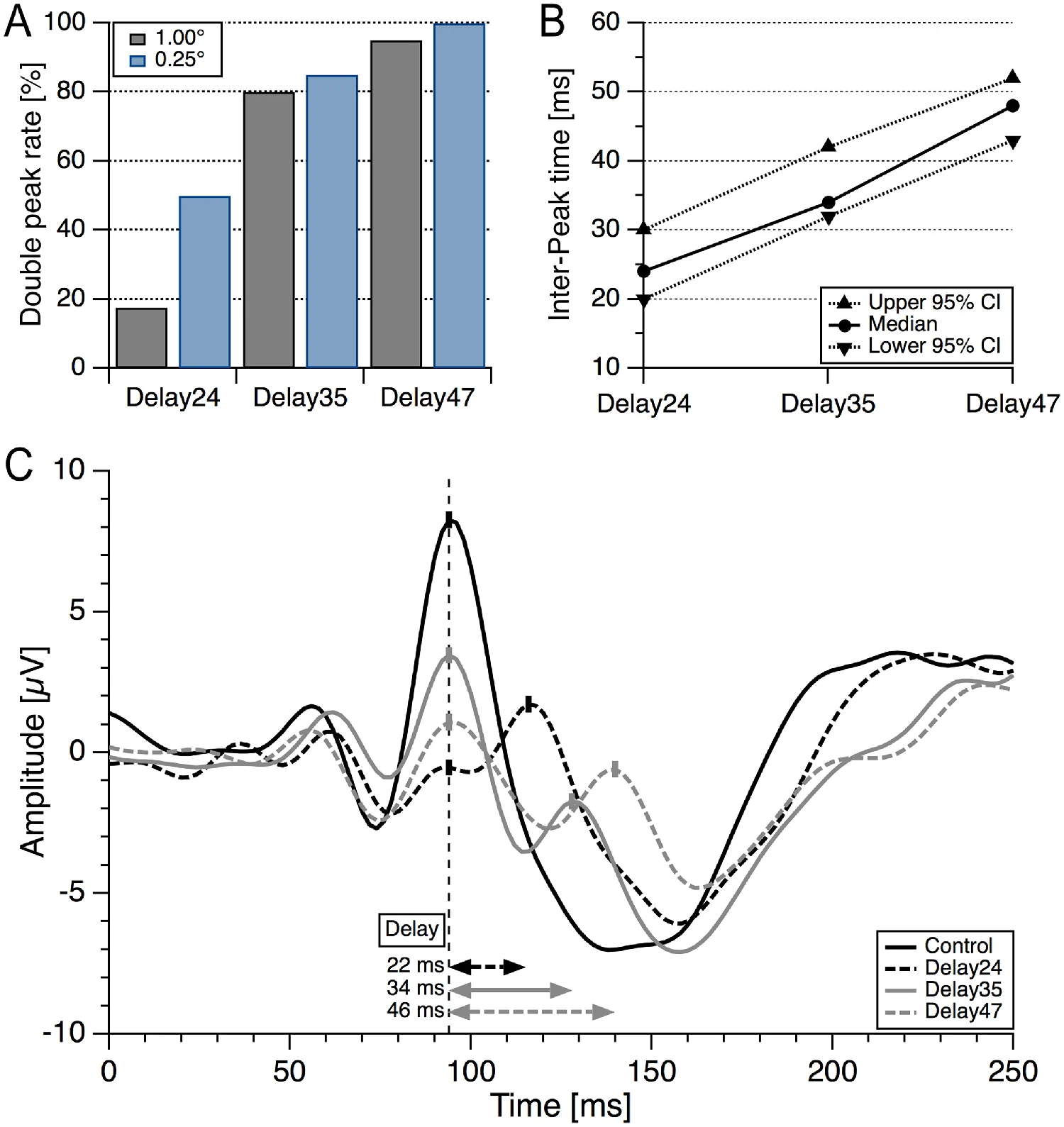
Double-peak incidence rates and inter-peak intervals across the delay conditions (*n* = 20). (**A**) Incidence for 1.00° (*black*) and 0.25° (*blue*) checks under Delay24, Delay35, and Delay47 conditions. (**B**) Median inter-peak intervals (peak time 2 − peak time 1) with 95% CIs for the 0.25° checks. (**C**) Waveforms from one participant (0.25° check size) with the first P100 peak of each delay condition aligned with the control peak in order to illustrate the atypical double peak morphology and the concurrent increase in inter-peak time with increasing reversal delays. *Arrows* at the base of the plot indicate the time difference between first and second peak for each delay condition.

The incidence of double peaks increased progressively with increasing half-field asynchrony, from 50% to 85% to 100% for Delay24, Delay35, and Delay47, respectively (Fig. 7A). There was a check-size dependence in the incidence with Delay24 (0.25°: 50% vs. 1.00°: 17.5%), which was absent at longer delays. Concurrently with the increasing reversal delay times, the median inter-peak intervals of the double-peaked P100 increased and closely mirrored the induced delays: 24 ms (95% CI, 20–30) for Delay24, 34 ms (95% CI, 32–42) for Delay35, and 48 ms (95% CI, 44–52) for Delay47 (Fig. 7B). This relationship is exemplified in Figure 7C.

**Figure 8.**
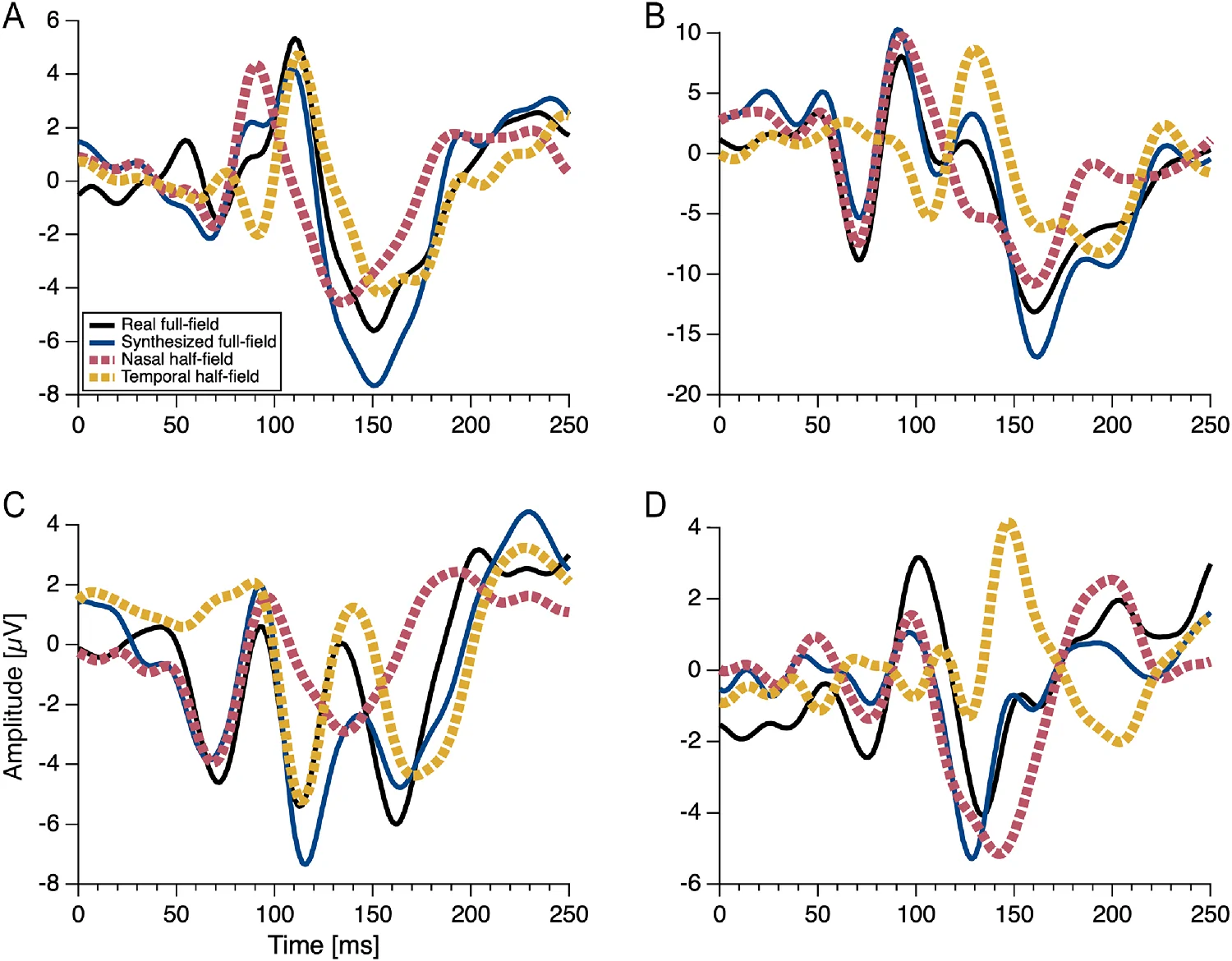
Waveforms of real (*solid black line*) and synthesized (*solid blue line*) *full-field* VEPs (asynchronous half-fields), alongside the respective undelayed nasal (*dashed red line*) and delayed temporal (*dashed yellow line*) *half-field* traces. (**A**) Shoulder on the ascending flank (24 ms delay). (**B**) Double peak (35 ms delay). (**C**) M-formation (47 ms delay). (**D**) Suppressed second peak (47 ms delay). Note the differences in ordinate scaling between panels.

Qualitative comparison of real and synthesized full-field VEP traces revealed a diverse range of morphologies that can be readily explained by the superposition of the undelayed nasal and synthetically delayed temporal half-field responses. Broadened complexes, plateau formations (Fig. 4), and shoulders (Fig. 8A, on the ascending P100 limb) occurred frequently with Delay24. Delay35 produced more pronounced shoulders and, more commonly, true double peaks such as the one visualized in Figure 8B. With Delay47, peaks were typically clearly separated, exhibiting an “M-shaped” configuration (Fig. 8C). However, occasionally the second peak could not be readily identified or was even completely suppressed, depending on the specific morphology of the half-field waveforms (Fig. 8D).

### Steady-State VEP

**Figure 9.**
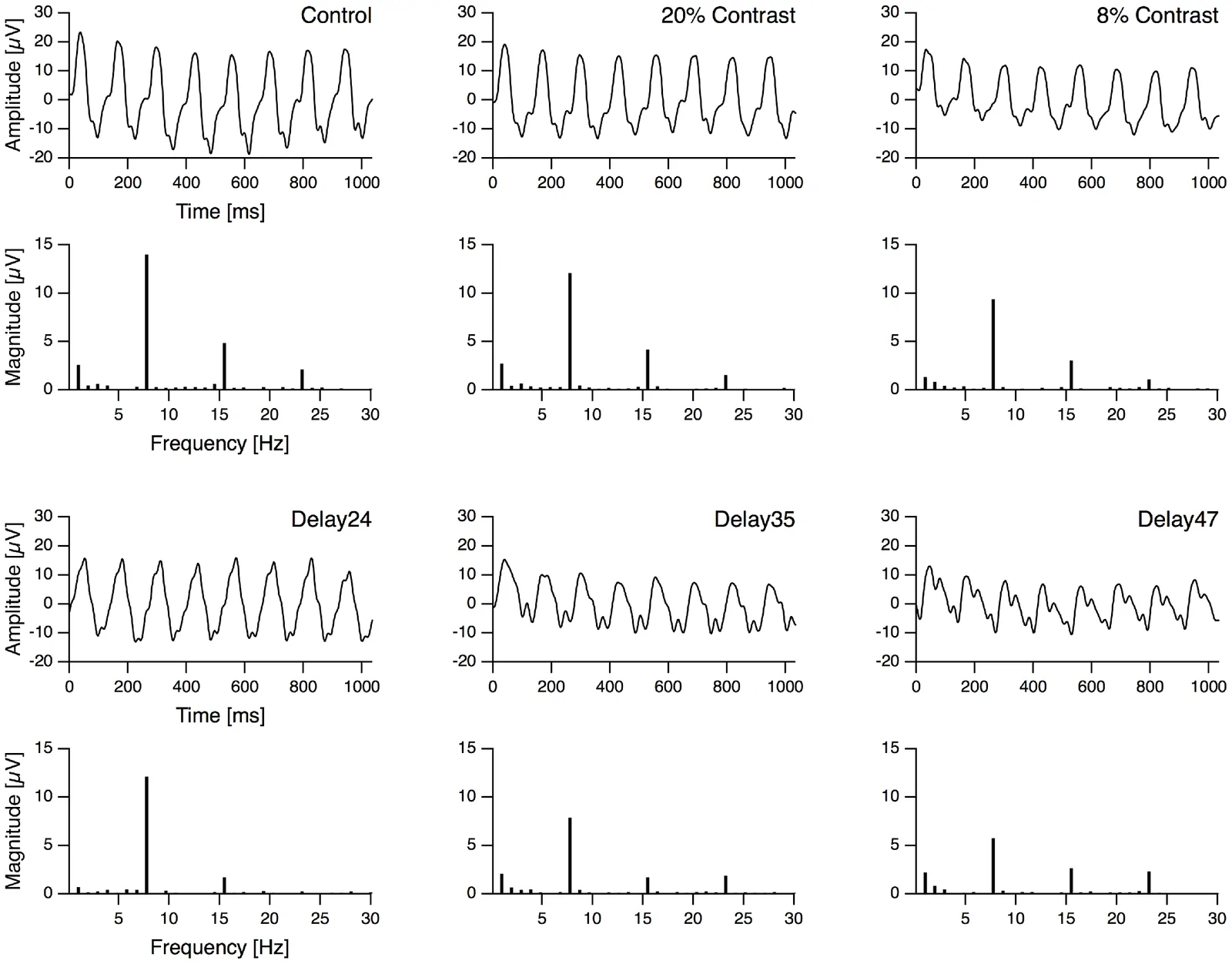
Example traces in the time and frequency domains from one participant for the full-field control, contrast, and delay conditions. Both the contrast and delay conditions exhibit an overall reduction of amplitude and Fourier magnitude. Critically, although the delay conditions also show pronounced changes in waveform morphology and frequency composition, these effects are not observed in the contrast condition.

As depicted in Figure 9, half-field contrast reduction led to a concurrent reduction in amplitude for both the first and second harmonics. In contrast, half-field asynchronies exerted a differential effect on the two harmonics. Delay24 and Delay35 induced an overall reduction but had a more profound effect on the second harmonic, causing the amplitude ratio (second-to-first) to decrease considerably. Delay47, in contrast, resulted in a marked increase in this ratio, implying that the second harmonic captured a larger fraction of the signal.

The following analysis focuses exclusively on the data from the Oz channel. Analysis of the virtual Laplace channel data demonstrated pronounced discrepancies between real and synthesized traces in both the time and frequency domains (Supplementary Fig. S2). Therefore we refrained from further analysis of these data. This finding is further addressed in the respective section of the discussion.

#### Control Condition

The Table presents the group means of the first-harmonic magnitude for the full-field traces for all three conditions, along with their corresponding 95% CIs, *P* values, and the ratio of the second to first harmonic. Nasal and temporal half-field traces demonstrated negligible differences in magnitude across all check sizes. Qualitative comparison of real and synthesized VEP traces in the time domain revealed good morphological agreement, while quantitative comparison in the frequency domain using Bland-Altman analysis yielded relative mean differences of −0.6 µV (1.00°), −2.4 µV (0.25°), and −0.8 µV (0.06°) (see Supplementary Material for plots).

**Table.** Group Level Mean Magnitudes of the First Harmonic and the Second-to-First Harmonic Ratios for the Control, Differential Contrast, and Delay Conditions

Condition	Mean Magnitude of First Harmonic [95% CI]	*P* Value	Second-to-First Harmonic Ratio	*P* Value
1.00°				
Control				
Real	4.7 µV [3.6–6.2]		40%	
Synthesized	5.2 µV [3.8–7.5]		40%	
Contrast				
20%	5.3 µV [3.9–7.3]	0.09	33%	0.25
8%	4.5 µV [3.3–6.1]	0.55	36%	0.50
Delay				
24 ms	4.4 µV [3.1–6.2]	0.51	32%	0.17
35 ms	3.7 µV [2.7–5.0]	<0.001	24%	0.02
47 ms	2.5 µV [1.8–3.4]	<0.001	59%	0.03
0.25°				
Control				
Real	9.2 µV [7.4–11.4]		28%	
Synthesized	11.5 µV [8.9–15.0]		24%	
Contrast				
20%	8.5 µV [6.9–10.8]	0.007	24%	0.13
8%	7.2 µV [5.8–8.7]	<0.001	22%	0.07
Delay				
24 ms	7.9 µV [6.1–10.1]	0.002	22%	0.08
35 ms	6.3 µV [4.9–8.0]	<0.001	22%	0.16
47 ms	4.1 µV [3.3–5.1]	<0.001	41%	0.08
0.06°				
Control				
Real	4.9 µV [3.9–6.1]		41%	
Synthesized	5.6 µV [4.5–7.1]		42%	0.33
Contrast				
20%	3.9 µV [3.2–4.7]	<0.001	38%	0.19
8%	2.9 µV [2.4–3.5]	<0.001	38%	0.19
Delay				
24 ms	4.0 µV [3.1–5.0]	0.005	28%	0.005
35 ms	3.3 µV [2.6–4.1]	<0.001	23%	<0.001
47 ms	2.3 µV [1.8–2.8]	<0.001	77%	0.03

Statistical significance relative to the control is indicated by *P* values.

#### Contrast Conditions

**Figure 10.**
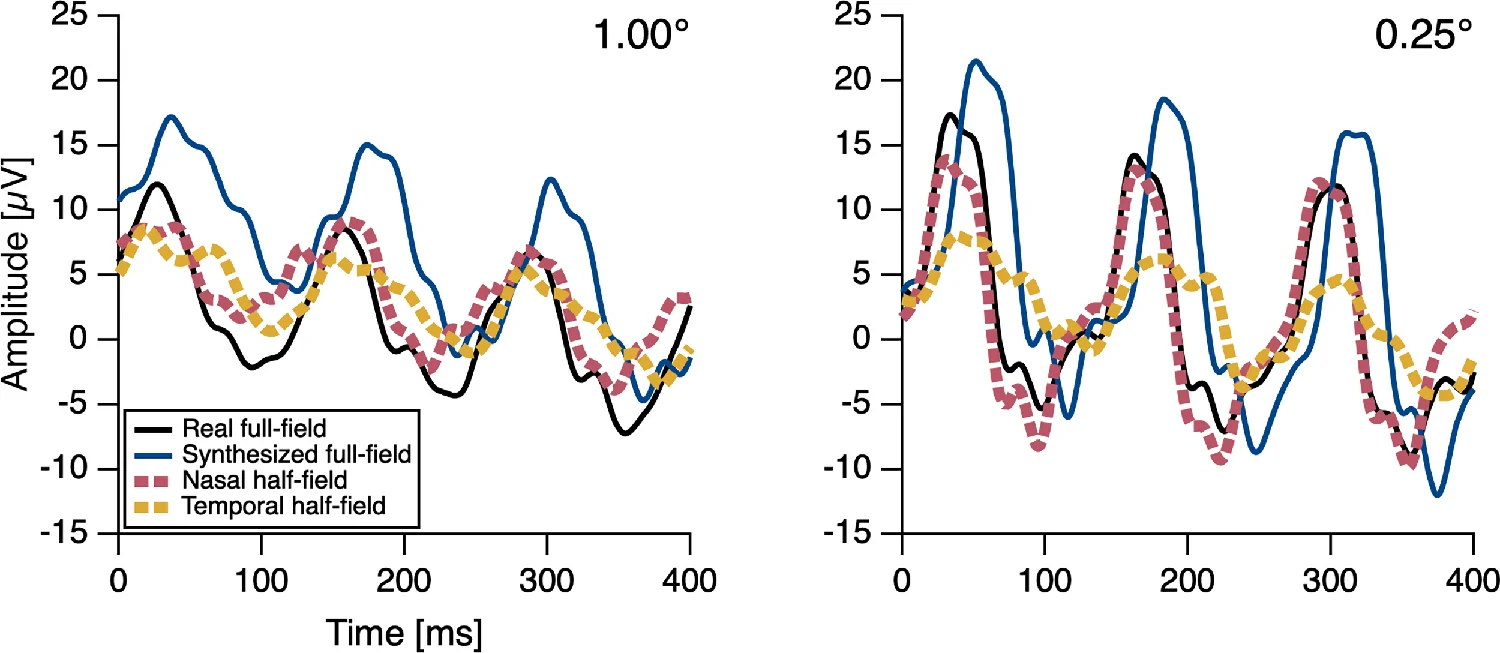
Example 1.00° and 0.25° 8% contrast traces from one participant showing real (solid black line) and synthesized (*solid blue line*) full-field, and nasal (*dashed red line*) and temporal (*dashed yellow line*) half-field traces. Contrast reduction demonstrates a check-size dependent effect, with a more pronounced reduction for the smaller check size. For visualization purposes, only 400 ms are shown.

Contrast reduction did not substantially affect the first harmonic magnitude for the full-field 1.00° check size. However, it significantly reduced magnitudes for 0.25° and 0.06° check sizes, particularly in the 8% contrast condition (Table). This is also evident when comparing the half-field waveforms and their amplitudes (Fig. 10). Critically, neither the second-to-first harmonic ratios (Table) nor the waveforms (Fig. 9) exhibited significant changes under contrast reduction.

#### Delay Conditions

**Figure 11.**
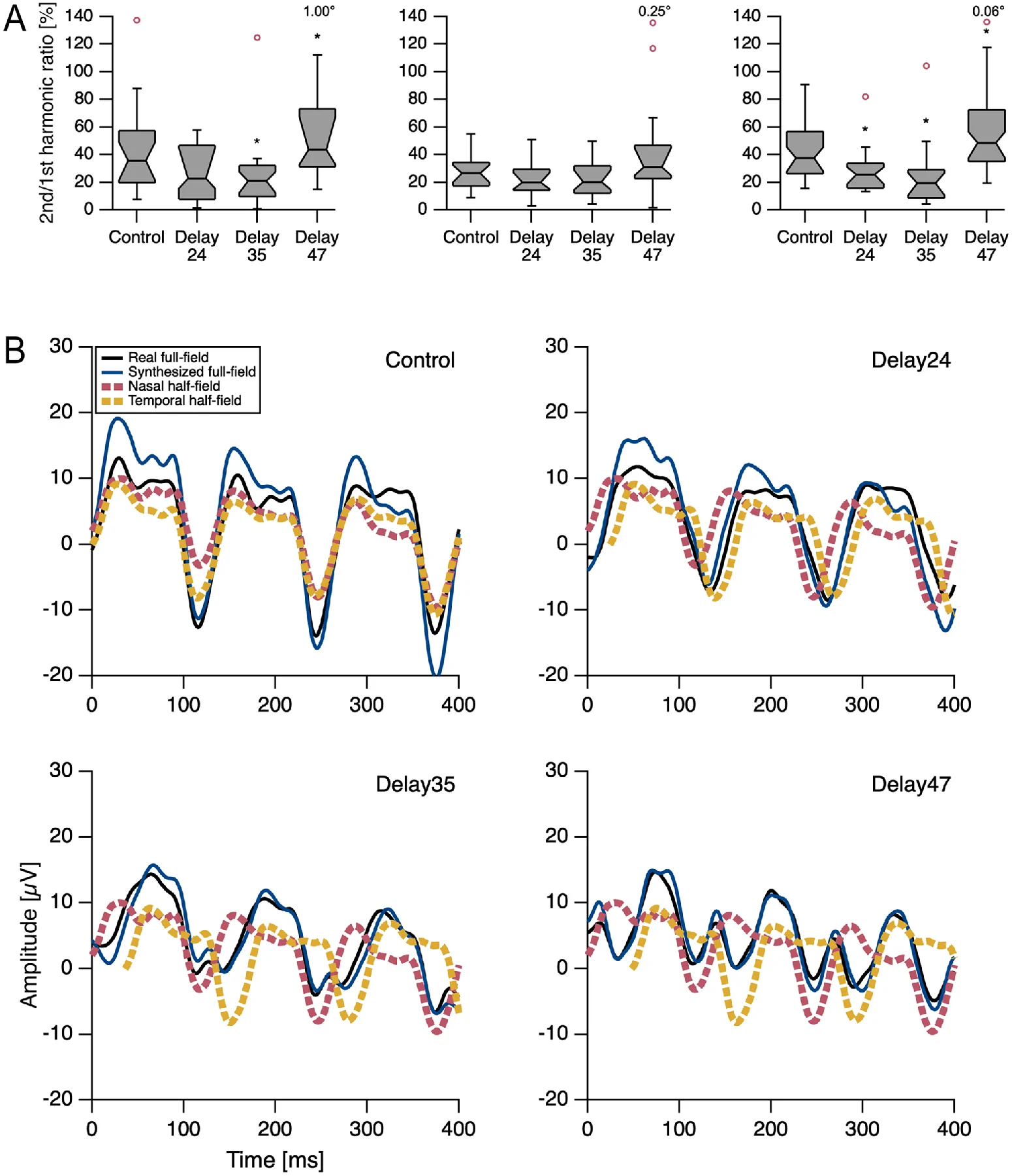
(**A**) Box plots of the second-to-first harmonic ratios [%] of all three check sizes and delays (*n* = 20). *Asterisks* mark statistically significant differences to the control. *Red dots* denote outliers. Note that two extreme outliers in the Delay47 condition (1.00°: 256%; 0.06°: 567%) are omitted from the plot for clarity. (**B**) Real (*solid black line*) and synthesized (*solid blue line*) full-field responses (asynchronous half-fields), alongside undelayed nasal (*dashed red line*) and delayed temporal (*dashed yellow line*) half-field traces from one participant for the 0.06° control and delay conditions. The second-to-first harmonic ratio tended to decrease under Delay24 and Delay35 but increased substantially under Delay47, particularly for the 1.00° and 0.06° stimuli. Synthesized full-field traces mostly align with the measured full-field responses. For visualization purposes, only 400 ms of the traces are displayed in **B**.

The mean magnitude of the first harmonic decreased progressively with increasing delay times for all three check sizes (Table). The second-to-first harmonic ratios were reduced for Delay24 and Delay35, and markedly increased for Delay47, relative to the control condition (Table; Fig. 11). Synthesized full-field waveforms illustrate the effects of constructive and destructive interference resulting from the superposition of time-shifted half-field responses. For instance, the characteristic waveform obtained with the longest delay results from constructive superposition of the shifted, plateau-like half-field responses at either end of the plateaus (see Fig. 11B: Delay47).

## Discussion

The present data are characterized by four main findings: (a) Asynchronous half-field stimulation successfully replicates the double-peaks and associated waveform features occasionally observed in clinical VEP recordings. (b) To a lesser degree, this holds true for differential-contrast half-field stimulation. (c) Synthetic full-field traces created by superimposing half-field recordings closely resemble real full-field traces under conditions of both synchrony and asynchrony, as well as with and without contrast differentials. (d) Large stimulus asynchronies in steady-state recordings increase the contribution of the second harmonic at the expense of the first harmonic. Taken together, this suggests a high plausibility for the assumption that conduction velocity differences explain at least some occurrences of double peaks and shoulder configurations in VEP recordings, particularly when supported by a broader clinical context compatible with optic nerve demyelination.

### Transient VEP

Consistent with prior experimental findings,^8^^,^^19^ our results support the fundamental assumption of the superposition principle in monocular VEPs. This confirms the additivity of half-field recordings for the subsequent qualitative and quantitative analysis of waveform morphology, double-peak formation and its underlying genesis. Although the present study focused on nasal and temporal half-field stimulation, the principle of superposition can be expected to apply to responses to upper and lower half-field stimulation, as demonstrated by Kessler and Heinrich.^20^ However, double peaks may manifest less clearly in this configuration because upper- and lower-field responses differ in waveform morphology and partly in polarity. Therefore partial conduction delays between upper and lower half-field responses may be less intuitive to interpret and could be more easily missed in the resulting full-field waveform. Responses to central versus peripheral field stimulation are equally expected to follow the basic principles of superposition. However, cortical folding differentially affects the scalp-measured responses to central and peripheral stimulation and often results in attenuation at higher eccentricities.^21^^–^^23^ A respective experimental design would thus introduce an additional source of variability and potentially compromise the signal-to-noise ratio while providing little benefit for the primary aim of the study. The choice of the nasal/temporal hemifield configuration was therefore made deliberately to achieve an optimal validation of the proposed superposition concept rather than to replicate details of the retinotopic distribution of conduction delays suspected in the disease.

Reduced stimulus contrast produced a robust, contrast-dependent decrease in P100 amplitude, accompanied by a small increase in P100 peak time and occasional double-peak appearances. Peak time delays remained below the practical threshold (>20 ms) proposed by an earlier theoretical model for reliable double-peak formation in the full-field response.^8^ This contrasts with the delay conditions, in which the imposed inter-half-field asynchronies were deliberately chosen to exceed the proposed threshold. Thus the large discrepancy in double-peak occurrence between the contrast and delay conditions is likely explained by the difference in half-field peak times. Qualitative analysis indicated that the overall amplitude reduction primarily originated from signal loss in the contrast-reduced half-field. Partial destructive superposition, caused by the temporal shift of the delayed half-field response, contributed only minimally to the overall amplitude loss.

Introducing asynchronies between stimulus half-fields revealed a clear positive relationship between the magnitude of delay and the likelihood of double-peak occurrence. The simulated delays closely corresponded to the measured inter-peak intervals, and a qualitative comparison showed that the P100 peaks of the individual half-field traces aligned temporally with the peaks of the full-field traces. Together, these findings suggest that in our experiment the two peaks in the full-field VEP represent temporally distinct P100 components reflecting cortical activation through functionally separate populations of optic nerve fibers. Although our experimental model does not provide a direct causal link between pathophysiology and VEP curve shape, it supports the plausibility of the idea that differentially affected nerve fiber bundles give rise to the double peaks found in clinical VEPs. The 24 ms delay condition, which produced an approximately equal probability of single- and double-peaked responses across participants, suggests that the emergence of double peaks is not bound to a strict temporal threshold but occurs within a range of inter-half-field delays. This range gives rise to a continuum of wave morphologies that evolves gradually with increasing interfield temporal delay, while the resulting waveform furthermore depends on individual VEP response characteristics.

Although our experimental model of delay is clearly a simplified representation of the pathophysiological reality in optic neuritis, it nonetheless provides a useful conceptual framework. Evidence from perimetric studies suggests that optic neuritis can affect specific retinal nerve fiber bundles, although patterns of involvement differ across studies and methodologies, with some reporting a predominance of localized macular deficits whereas others suggest a more diffuse visual field impairment.^22^^–^^24^ For the case of localized macular involvement in optic neuritis, Rinalduzzi et al.^25^ showed that the P100 mediated by macular fibers is considerably more delayed compared to peripheral fibers. In agreement with this, Brecelj et al.^26^ linked W-shaped responses to impaired macular fiber function, although these were not consistently associated with a central field defect. Despite this latter inconsistency, the central-versus-paracentral manifestation likely represents a common clinical variant of the superposition mechanism examined here. Irrespective of the above-mentioned challenges, an extension of the present study to this retinotopic configuration might prove particularly informative, especially considering the known interaction between checker size and eccentricity in VEP responses,^27^ which may modulate double peak appearance.

From a theoretical point of view, optic neuritis-related VEP changes may be considered to occur along a spectrum of conduction-related waveform alterations, including relatively isolated P100 peak-time prolongation, variable degrees of waveform broadening, shoulder-like configurations, and, less commonly, double-peaked responses. Clinically, the most common sustained finding after an episode of optic neuritis is P100 peak-time prolongation, which may occur with only minor waveform changes or with variable degrees of peak broadening. A delayed but relatively well-defined P100 response could be compatible with comparatively homogeneous slowing of conduction across the dominant response-generating fiber population. A broadened P100 response may reflect greater temporal dispersion, possibly due to more gradual conduction delays across multiple fiber populations. However, such interpretations should remain cautious, because waveform morphology does not allow a straightforward inference regarding the underlying pattern of fiber involvement. Although a broad peak can result from gradual conduction dispersion across a continuously affected fiber population, our data also show that separate delayed response components do not always produce two distinct peaks; depending on their timing and relative amplitudes, they may instead appear as a broadened peak or a shoulder-like waveform. Conversely, when delayed components remain sufficiently well-defined and temporally separated, a double-peaked configuration may become apparent. Thus the variety of waveform configurations may be viewed as related outcomes along a continuum of conduction asynchrony rather than as mutually exclusive signatures of gradual or discrete fiber involvement.

Within this framework, our findings do not imply that double peaks are a prevailing manifestation of optic neuritis-related demyelination nor that they provide direct evidence for a specific anatomical pattern of involvement. Rather, they support the more specific conclusion that differences in conduction timing between functionally separable nerve fiber populations are sufficient to generate waveform configurations resembling those occasionally observed in clinical VEP recordings. This interpretation is compatible with the clinical observation that optic neuritis may result in substantially delayed yet relatively well-defined P100 responses. Our model uses this established observation and demonstrates how the superposition of two such response components, when affected to different degrees, can give rise to a range of waveform configurations, including broadening, shoulder-like responses, and double peaks. The sparse functional data from visual field testing and multifocal VEP studies in optic neuritis^28^^–^^30^ point toward a spatially non-uniform involvement of retinal nerve fiber bundles, thereby tentatively supporting the assumptions underlying the response-component interactions proposed here. Although clinical complexity and various factors influence VEP morphology, this interpretation provides a compelling framework for further validation through spatially resolved functional testing, such as mfVEP or perimetry, particularly in cases involving double peaks or pronounced waveform broadening.

Beyond our proposed mechanism, several others have been proposed in the literature to produce double-peaked P100 signals. First, interindividual variation in the area and location of the visual cortices relative to standard craniotopic landmarks may produce unusual field distributions and account for double-peaked responses in healthy individuals.^6^ Second, a temporal dissociation of the frontal N100 signal from the occipital P100, with detection of the former at reversed polarity, has also been suggested as a source.^31^ Third, the double-peaked P100 appearance has also been attributed to interference of the polarity-reversed P135 signal from the contralateral secondary visual cortex and associated with macular visual field defects.^32^ Last, it is known that retinal diseases, for instance those affecting the macula,^33^ may result in delayed VEP responses. It is conceivable that differential impairment of the retina might in some cases result in VEP double peaks. Taken together, these alternative mechanisms further support the notion that double peaks need to be interpreted in the context of other clinical findings.

### Steady-State VEP

Qualitative comparison of real and synthesized ssVEP traces demonstrated a high degree of concordance, although some inter-subject variability was observed. This qualitative impression was supported by quantitative analyses, which showed moderate agreement that was most robust for the largest check sizes but less pronounced for smaller stimuli.

Contrast reduction had a check-size-dependent effect: mid-range sizes (1.00°) were largely unaffected, consistent with the notion of a negative notch for high-contrast patterns.^12^^,^^34^ Conversely, smaller check sizes (0.25° and 0.06°) showed a progressively reduced first harmonic magnitude, reflecting predominant amplitude modulation with little temporal variation in the half-field responses. Correspondingly, the second-to-first harmonic ratio remained largely unchanged across contrast levels.

The effects on waveform and harmonic content varied across the different delay manipulations. The two shorter delays (24 ms and 35 ms) reduced overall amplitude, with a pronounced decrease in the second-to-first harmonic ratio, reflecting more sinusoidal waveforms resulting from partial destructive interference of the broad positive half-field plateaus. At the longer delay (47 ms), waveforms became jagged and multi-peaked, with increased second-to-first harmonic ratios, largely independent of check size. This shift in harmonic composition might cause misleading results, especially in those cases where only a single harmonic is evaluated.^35^ For interpretation, it is important to note that higher harmonics should not be understood as separate physiological processes. Rather, they reflect the frequency-domain representation of the recorded waveform's temporal structure. Accordingly, the increased contribution of the second harmonic in the present study corresponds to changes in waveform shape, including more complex or multi-peaked responses, rather than a distinct physiological generator at the second harmonic frequency. Importantly, the observed effects on harmonic ratios represent a novel finding and, alongside previously reported phase shifts,^36^^,^^37^ might inform the interpretation of steady-state data, including those obtained in the context of objective acuity testing.

Finally, it should be noted that the agreement between real and synthesized ssVEP traces was less clear in the virtual Laplace channel data, where pronounced discrepancies were observed between the real and synthesized full-field VEPs in both the time and frequency domains. A more comprehensive understanding of this mismatch is required before this modeling approach can be robustly applied to Laplacian data; consequently, these traces were excluded from the present analysis.

## Conclusions

Our findings suggest timing asynchronies to be a plausible cause of P100 double peaks in optic neuritis. Our experimental model successfully replicated such double peaks through temporal differences in the stimulation of different parts of the visual field. In clinical cases, we assume that double peaks may arise from differences in conduction speed between distinct nerve fiber bundles, likely due to focal changes of myelination during the inflammatory or recovery phase. The findings further substantiate the argument by Morny et al.^8^ that VEP assessment in suspected optic neuritis should consider more than just the timing of the largest peak.

## Supplementary Material

Supplement 1
