# Supplementary material for: Simulating Partial Conduction Delays in VEPs Via Asynchronous and Contrast-Modulated Stimulation
Source: Transl Vis Sci Technol. 2026 Sep 8;15(9):5. doi: 10.1167/tvst.15.9.5 (PMC13574218; doi:10.1167/tvst.15.9.5)
Supplement: Supplementary file 1 [file tvst-15-9-5_s001.pdf]

Supplementary data

Table S1: Characteristics of study populations. VA = visual acuity, CS = contrast sensitivity, OD = *oculus dexter*/right eye, OS = *oculus sinister*/left eye; *n* = 20 per column.

	trVEP	ssVEP
Mean age (range)	26.3 years (19 – 44)	26.4 years (19 – 44)
Gender (M/F)	7/13	5/15
Mean VA (range)	−0.23 (−0.02 – −0.38)	−0.24 (−0.01 – −0.39)
Mean CS (range)	1.70 (1.61 – 1.82)	1.66 (1.60 – 1.82)
Recorded eye (OD/OS)	13/7	9/11

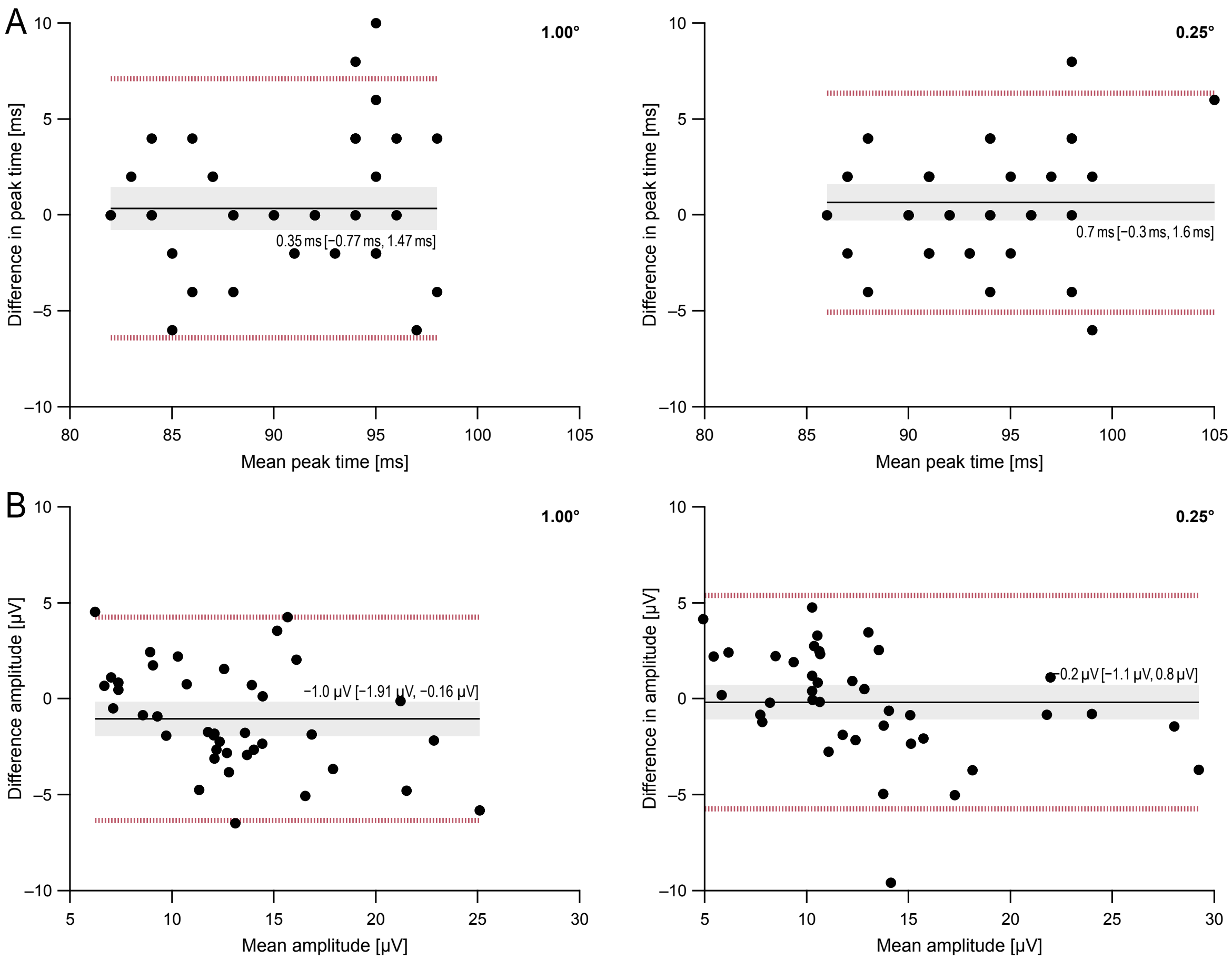

Figure S1: Bland-Altman plots of real and synthesized P100 peak time (A) and amplitude (B) for the trVEP 1.00° and 0.25° full-field control conditions (*n* = 20). Abscissa: mean of the paired data points from the real and synthesized data sets. Ordinate: difference between the corresponding data points (real minus synthesized). The solid black line indicates the mean difference, with the grey bar representing the ±95% CI. Red lines correspond to the lower and upper limits of agreement.

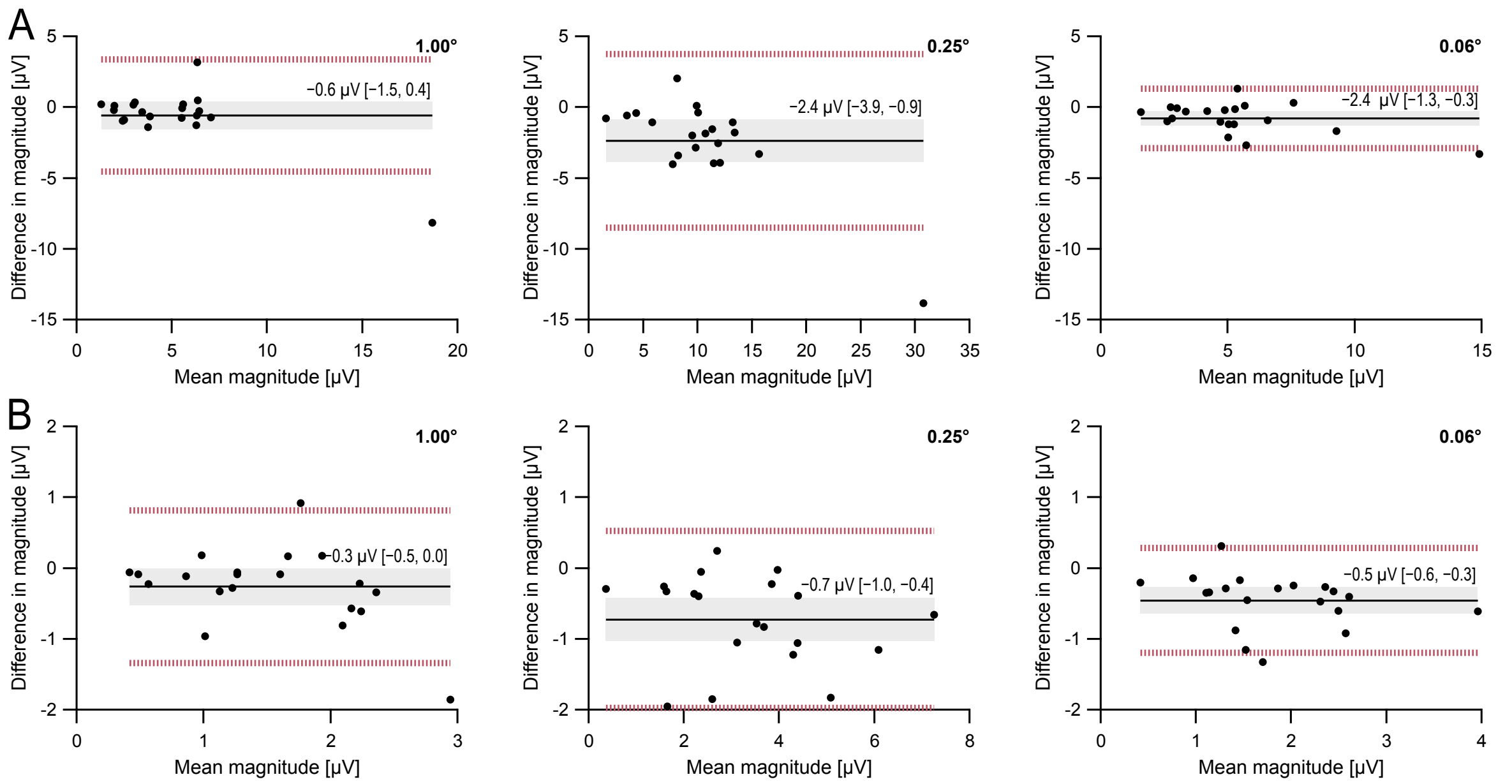

Figure S2: Bland-Altman plots of real and synthesized first harmonic magnitudes for the 1.00°, 0.25°, and 0.06° full-field control conditions (*n* = 20). (A) Data from the Oz recordings; (B) data from the virtual Laplace channel (Oz−(O1+O2)/2). Abscissa: mean of the paired data points from the real and synthesized data sets. Ordinate: difference between the paired data points (real minus synthesized). The solid black line indicates the mean difference, with the grey bar representing the ±95% CI. Red lines correspond to the lower and upper limits of agreement. Note the differences in ordinate scaling.
